# The Molecular Mechanisms in Senescent Cells Induced by Natural Aging and Ionizing Radiation

**DOI:** 10.3390/cells13060550

**Published:** 2024-03-21

**Authors:** Milana Ibragimova, Assiya Kussainova, Akmaral Aripova, Rakhmetkazhi Bersimbaev, Olga Bulgakova

**Affiliations:** 1 Department of General Biology and Genomics, Institute of Cell Biology and Biotechnology, L.N. Gumilyov Eurasian National University, Astana 010008, Kazakhstan; milanaibragimova2602@yandex.ru (M.I.); assya.kussainova@gmail.com (A.K.); aripova001@gmail.com (A.A.); ribers@mail.ru (R.B.); 2 Department of Health Sciences, University of Genova, Via Pastore 1, 16132 Genoa, Italy

**Keywords:** cellular senescence, natural aging, ionizing radiation, DNA damage, mitochondrial dysfunction, miRNAs, SASP

## Abstract

This review discusses the relationship between cellular senescence and radiation exposure. Given the wide range of ionizing radiation sources encountered by people in professional and medical spheres, as well as the influence of natural background radiation, the question of the effect of radiation on biological processes, particularly on aging processes, remains highly relevant. The parallel relationship between natural and radiation-induced cellular senescence reveals the common aspects underlying these processes. Based on recent scientific data, the key points of the effects of ionizing radiation on cellular processes associated with aging, such as genome instability, mitochondrial dysfunction, altered expression of miRNAs, epigenetic profile, and manifestation of the senescence-associated secretory phenotype (SASP), are discussed. Unraveling the molecular mechanisms of cellular senescence can make a valuable contribution to the understanding of the molecular genetic basis of age-associated diseases in the context of environmental exposure.

## 1. Introduction

Cellular senescence is a state of irreversible cell growth arrest mediated by multiple factors. Senescence is known to increase the risk of cancer and other pathologies associated with aging, including neurodegenerative diseases [1]. This is the reason for the increased attention given by the scientific community to the molecular mechanisms of aging.

Every day, cell integrity is threatened not only by endogenous agents, but also by exogenous factors, among which radiation exposure occupies a key position. Recently, radiation has become widely associated with a spectrum of age-related diseases, noting that ionizing radiation (IR) itself may be a cause of premature aging [2]. Exposure to IR causes several physiological changes that can lead to DNA double-strand breaks (DSBs), thereby initiating genomic instability and metabolic system dysfunction [3]. The impact of IR can vary significantly, influenced by a range of factors such as the radiation dose. High doses of radiation can cause acute radiation sickness, whereas prolonged low-dose exposure often leads to chronic pathologies [4].

Some studies have shown that low-dose irradiation, acting on bone marrow mesenchymal stromal cells (BMSCs), promotes failure of cellular processes. In particular, IR initiates cell cycle arrest, disrupts autophagy, and accelerates senescence [5]. These results are consistent with the work of Wang et al. [6], who demonstrated that after IR exposure, there is an accumulation of senescent cells, an increase in senescence biomarkers in radiation ulcers, and the development of senescence-associated secretory phenotype (SASP). This shows a non-targeted effect of radiation mediated by the paracrine abilities of senescent cells to initiate senescence, inflammation, and SASP in neighboring cells not directly exposed to radiation. Moreover, IR has been reported to exacerbate carotid atherosclerosis by inducing plaque growth, persistent DNA damage, overexpression of inflammatory genes, SASP development, and cellular senescence [7].

The biological effects of IR are not limited to DNA damage and inflammation. Radiation exposure directly affects the mitochondria by inducing mitochondrial dysfunction through increased levels of reactive oxygen species (ROS), mediating disruptions in the electron transport chain (ETC), and oxidative stress [8]. IR-induced oxidative stress is known to have a deleterious effect on telomeres, thereby accelerating their shortening in cells [9]. ROS-mediated DNA damage accumulates over time, leading to irreversible changes in the structural and functional integrity of cells, which are associated with age-related phenotypes [10]. In addition, senescent cells tend to accumulate dysfunctional mitochondria, which increases ROS production and induces SASP [11].

To date, the study of radiation-induced cellular senescence is increasing, owing to the increased exposure of humans to IR in many areas of life. The age at the time of exposure is an important factor in radiation-induced cellular senescence. In a study by Hoyes et al. [12], γ-irradiation was shown to initiate bone marrow stem cell deficiency in mice at different stages of development; however, the embryonic period exhibited the highest radiosensitivity. In general, it has been observed that individuals exposed early in life show increased radiosensitivity, which gradually decreases and then increases with age [13]. The excess relative risk (ERR) is higher in youth exposed to radiation, which also suggests hypersensitivity to radiation during these years [14].

This review discusses the general molecular mechanisms that initiate natural- and radiation-induced cellular senescence and recent research in the field of studying the effects of IR on senescence processes. Understanding the senescence pathways and the role of radiation in them will allow the development of therapeutic methods to slow down aging and minimize the negative effects of radiation exposure.

## 2. Inducers and Key Signatures of Cellular Senescence

Natural cellular senescence is defined by multiple features, among which the key focus is on genome instability, mitochondrial dysfunction, specific miRNA expression, altered epigenetic profile, and SASP acquisition [1,4,15].

Nevertheless, in the issue of inducers, we should additionally mention one of the first and most well-characterized mechanisms of cellular senescence, telomere shortening [16], as well as the first widely used biomarker for the detection of senescent cells, senescence-associated β-galactosidase (SA-β-gal) [17]. There has been an extensive amount of research, including in the last few years [18,19,20,21], aimed at investigating the mechanisms of senescence and ways to influence them, using SA-β-gal as a key senescence marker.

Telomere shortening, a natural occurrence with each cell division, restricts the replicative capacity of cells, which is also known as replicative senescence [22]. As early as 1961, Hayflick et al. [23] determined that mammalian cells have a replicative capacity, referred to as the “Hayflick limit” and equal to 40–60 cycles. When reached, irreversible cell cycle arrest is initiated, directly leading to replicative senescence. 

### 2.1. Genome Instability

DNA damage is a significant marker of cellular senescence, and maintaining genome stability is of paramount importance to organisms. This has led to the evolutionary development of complex DNA repair mechanisms commonly known as the DNA damage response (DDR) [13,24].

DNA double-strand breaks (DSBs) are a very dangerous type of damage that most often leads to mutations, subsequently initiating apoptotic death and neoplastic transformation [24]. When DSBs occur in the cell, the DDR pathway, which consists of multiple proteins, is activated [25]. DSBs are first recognized by sensor proteins, such as the MRE11/RAD50/NBS1 (MRN) complex, their recruitment occurs [26], and the break information is passed to ATR/ATM kinases [27], involved in the phosphorylation of histone H2AX (γH2AX) [28,29]. The activation of ATM through MRN-dependent recruitment results in its autophosphorylation on S1981. ATM phosphorylates the histone H2AX on its C-terminal tail on serine 139. γH2AX functions as a docking site for the recruitment of the mediator MDC1 on the chromatin flanking the DSB, which amplifies the DDR signaling cascade [30].

Ultimately, activation of the DDR system triggers a cascade of different cellular pathways, in which p53, p21, and p16 are key players [13,24,31]. DDR controls the cell cycle by stopping it to allow damaged DNA to be repaired; if this is no longer possible, the cell dies by apoptosis or can lead to senescence [24,26,32]. Several signal transduction pathways of the DDR complex are the major drivers of senescence, including the p53–p21 pathway required for senescence induction and p16–pRb signalling, which serves to maintain senescence [31]. 

After successful DNA repair, DDR foci disappear after some time [33]; however, in the case of irreversible damage, persistent DDR foci remain, which can initiate the cell’s transition into a senescent state [25]. DSBs formed in telomeric sequences play a particularly important role in cellular senescence. As telomeric DSBs are difficult to repair [34], they can accumulate over time, as evidenced by the prolonged presence of γH2AX foci in telomeres [35]. Moreover, there is evidence that age-related telomere shortening is positively correlated with the formation of persistent DDR foci [16,25]. The senescent phenotype develops slowly within days of the initial disruption of DNA integrity and is maintained by continued activation of the DDR system [29]. Activated DDR components are used as markers of DNA damage, the most sensitive of which is phosphorylated histone H2AX [36]. Increased levels of γH2AX may indicate replicative senescence and premature aging induced by exogenous sources of DNA damage such as IR [28,33]. Thus, the duration of mesenchymal stem cell (MSC) cultivation correlates with the ATM-dependent accumulation of γH2AX foci [37].

Although most DNA damage is successfully repaired, some is erroneously repaired or appears irreparable, which can lead to serious consequences including apoptosis, cellular transformation, or direct induction of cellular senescence [31,38]. This is partly due to age-related decline in the performance of repair systems [39]. It has been observed that the accumulation of microstructural chromosomal abnormalities resulting from defective repair of DSBs plays a fundamental role in cellular senescence [40]. Some authors consider the accumulation of DNA damage in cells and dysfunction of the repair machinery [41,42] as causes of premature aging syndromes (progeroid syndromes), noting that the genetic mutations underlying them are functionally related to genome stability [43]. Thus, DNA damage inevitably accumulates over time, making genomic instability a characteristic feature of cellular senescence. 

### 2.2. Disruption of Mitochondrial Profile

The disruption of mitochondrial function has significant implications for cell cycle regulation, disease pathogenesis, and cellular senescence. Disruption of mitochondrial structural and functional integrity is considered to be one of the main causes of cellular senescence, mediating oxidative damage of mitochondrial DNA (mtDNA), increased ROS production, and decreased antioxidant defense of the organism [44]. In general, natural aging itself is accompanied by a general decline in mitochondrial quality and activity in all tissues [45], which is mediated by the accumulation of mtDNA mutations and increased ROS production [46].

The quantitative ratios of NAD+/NADH and NADP+/NADPH are crucial for major cellular processes, including DNA repair and epigenetic regulation [47]. With age, there is an increase in the reduced forms of NADH and NADPH and a decrease in NAD+ and NADP+ [47]. It has been noted that age-associated loss of NAD+ may be associated with imbalances in metabolic system function, chronic inflammation, and DNA damage [48,49]. Another reason for age-related decline in NAD+ levels in the body may be the low rate of NAD+ biosynthesis. NAMPT and NAPRT are enzymes that catalyze NAD+ biosynthesis from its precursors [50]. The age-related decrease in NAMPT expression leading to decreased NAD+ levels has been identified as a key factor [51]. Increasing NAD+ levels are a promising strategy to combat age-related dysfunction [52]. Low-dose nicotine, a secondary metabolite of NAD+ biosynthesis, has been shown to restore age-related decline in NAMPT activity by binding to SIRT1 and subsequently deacetylating NAMPT, leading to increased NAD+ synthesis and improved cellular energy metabolism. Nicotine has various positive effects on the body, including stimulating neurogenesis and reducing neuroinflammation in aged mice and protecting organs from damage caused by oxidative stress and telomere shortening [53].

Cellular senescence involves the accumulation of dysfunctional mitochondria, decreased ATP production, increased ROS levels, and impaired antioxidant systems. High levels of ROS are known to lead to oxidative stress and induce oxidative DNA damage [44,54], which activates the DDR pathway to restore structural integrity. However, it can be disrupted in a chronic oxidative environment, leading to the accumulation of persistent DDR foci [55]. The level of 8-hydroxy-2′-deoxyguanosine (8-OHdG) has been used as a key biomarker of oxidative DNA damage [56,57].

mtDNA is a vulnerable and well-detected target for the detection of many pathologies. Moreover, the close proximity of ROS and mtDNA production sites should be considered, as they contribute to the accumulation of oxidation-induced DNA damage [54]. Accumulation of mtDNA mutations leads to changes in mitochondrial biogenesis and function, which can initiate premature cellular senescence and the manifestation of an age-related phenotype [58,59,60,61]. Somatic mtDNA mutations accumulate in the stem cell population and contribute to mitochondrial dysfunction [62]. The mtDNA deletion is Δ-mtDNA^4977^, the most commonly considered a somatic mutation, which increases with age during the progression of mitochondrial dysfunction [63]. Free-circulating mtDNA (cf-mtDNA) is another favored non-invasive biomarker for multiple diseases; for example, it has been used to diagnose various cancers [64,65,66,67]. Recently, cf-mtDNA has been used as a predictive marker of the age-related phenotype [68,69,70].

Mitophagy is another key factor involved in cellular senescence. Mitophagy controls mitochondrial quality by selectively destroying defective organelles. A decrease in membrane potential indicates impaired mitochondrial functional integrity [71]. Increasing evidence suggests that mitophagy is impaired in pathological conditions [72]. In mammals, the mitophagy pathway includes the mitochondrial kinase PINK1 and E3 ubiquitin ligase Parkin, which undergo posttranslational modifications [73]. PINK1 avoids proteolytic degradation by accumulating on damaged mitochondria, where it phosphorylates ubiquitin on outer membrane proteins. These phospho-ubiquitin chains then bind to Parkin, recruiting it from the cytosol to the mitochondria and activating its E3 ubiquitin ligase activity [45].

In a study by Ahmad et al. [74], stress-induced cellular senescence in lung fibroblasts induced by the action of cigarette smoke extract (CSE) demonstrated functional impairment of mitochondria, manifested by an increase in mitochondrial ROS (mtROS) and a decrease in ATP levels, as well as structural alteration of organelles. After CSE exposure, there was an increase in mitochondrial mass and a strong elongation and accumulation of mitochondria in the perinuclear space, leading to an increase in ROS levels and the formation of γH2AX foci. Perinuclear mitochondrial accumulation is a signal for mitophagy; however, CSE treatment stabilizes PINK1 and disrupts Parkin translocation, indicating that mitophagy is impaired.

Increased oxidative stress occurring under the action of exogenous and endogenous agents in senescent cells is associated with the accumulation of dysfunctional mitochondria and the development of a specific secretory phenotype [16]. Wiley et al. [75] defined mitochondrial dysfunction as the key reason for the formation of a specific phenotype—“mitochondrial dysfunction-associated senescence” (MiDAS). The authors noted that MiDAS appears to be less related to DNA damage accumulation or ROS than mediated by a decrease in the NAD+/NADH ratio leading to AMP-activated protein kinase (AMPK) activation, which may initiate cellular senescence by phosphorylation of p53.

Sirtuins (SIRTs) are involved in mitochondrial function. SIRTs are thought to exert mitochondrial quality control by mediating mitophagy [76]. The mitochondrial sirtuins SIRT3, SIRT4, and SIRT5 function as links between aging and cell metabolism [77]. Decreased SIRT activity and NAD+ levels with age are involved in the pathogenesis of a wide range of cardiovascular and metabolic diseases including atherosclerosis, endothelial dysfunction, acute cardiac syndromes, arrhythmias, hypertension, diabetes mellitus, and dyslipidemia [78]. 

### 2.3. miRNAs in the Mechanism of Cellular Senescence

MicroRNAs (miRNAs) are small noncoding RNAs involved in the regulation of gene expression at the posttranscriptional level [79]. miRNAs control the transition from replicating to senescent cells by targeting factors that respond to cellular stress and different signaling pathways [80]. miRNAs modulate cellular senescence by regulating signalling proteins, particularly p53/p21/p16/Rb, which potentially promote or delay senescence [81]. 

An extensive number of miRNAs involved in senescence processes have been reported, which are tentatively summarized under the term “senescence-associated microRNAs” (SA-miRNAs) (Table 1). Altered expression of SA-miRNAs initiates DNA damage, γH2AX focus formation, ROS increase, and oxidative stress, supporting an age-related phenotype [82].

miR-34a, a key regulator of *SIRT1*, is one of the major miRNAs involved in cellular senescence [83]. A positive feedback loop exists between miR-34a, *SIRT1*, and p53 where p53 activates miR-34a expression, which targets and represses *SIRT1*, preventing *SIRT1*-mediated deacetylation of p53, thus promoting p53 activity [80]. Overexpression of miR-34a antagomir in primary epithelial cells from COPD patients decreased miR-34a levels and p21 and p16 expression and increased *SIRT1* mRNA expression [100]. These data suggested that miR-34a is an important regulator of cellular senescence.

The expression level of miR-21 is increased in senescent liver endothelial cells (HLSECs) and human fibroblasts, including stress-induced senescence, which suggests a role for miR-21 as a key player in the induction or maintenance of cellular senescence [86]. Mice with miR-21 knockout show resistance to D-gal-induced cardiac alterations, and on the contrary, increased miR-21 expression can stimulate cardiomyocyte senescence induced by doxorubicin (Dox) treatment [101]. 

There are contradictory data regarding the role of miR-146a expression in cellular senescence. Vasa-Nicotera et al. [87] observed a decrease in miR-146a expression levels in senescent umbilical vein endothelial cells (HUVECs). miR-146a targets *NOX4 (NADPH oxidase 4)*, which is the major source of ROS in these cells. In contrast, Deng et al. [88], studying the molecular mechanisms underlying the senescence of lineage negative bone marrow cells (lin-BMCs), found that miR-146a is overexpressed in senescent lin-BMCs, regulating cellular senescence and apoptosis by suppressing *Plk2* expression and activating p16^Ink4a^/p19^Arf^ and p53. 

Analysis of miRNAs profiles during induced cellular senescence in MSCs identified 21 miRNAs with increased expression levels, including miR-29a-3p, miR-34a-5p, miR-22-3p, miR-30d-5p, miR-22-5p, and miR-125b-5p [102]. Members of the miR-29 and miR-30 families are involved in the induction of senescence. During cellular senescence, Rb-dependent overexpression of miR-29 and miR-30 occurs, which was confirmed by a decrease in their levels in HeLa/E6 cells in response to Rb family knockout [89]. 

Extracellular vesicles (EVs) mediate intercellular communication and play an important role in cellular senescence [103]. Significantly increased levels of miRNAs of the miR-29 family in EVs control aging processes in adipose tissues of mice. A similar trend was observed for miR-146-5p [104]. The expression of miRNAs in EVs was increased in senescent fibroblasts: miR-29a-3p, miR-34a-5p, miR-30a-3p, miR-24a-3p and miR-186-5p [105]. 

**Figure 1:**
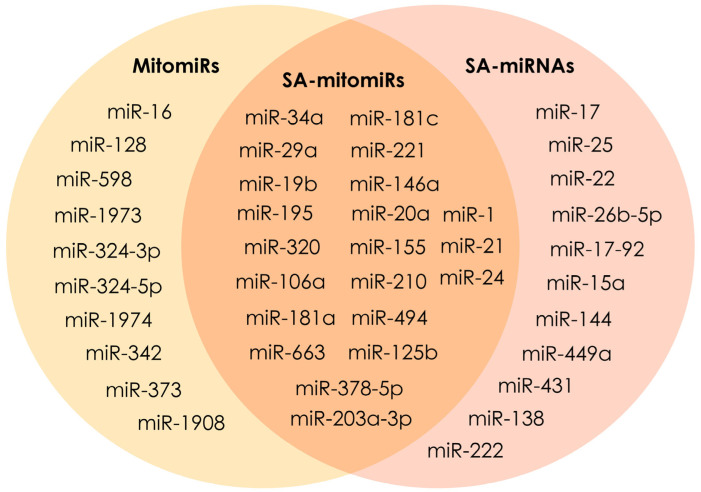
Venn diagram showing the mitomiRs involved in senescence (SA-mitomiRs). MitomiRs and SA-miRNAs are grouped according to the study in which they were described. Shared SA-mitomiRs are shown in the intersections.

The previously mentioned mitochondria have been determined to be directly involved in cellular senescence, but another interesting approach is to study the role of mitochondrial miRNAs, grouped under the term “mitomiRs”, in senescence processes (SA-mitomiRs) (Figure 1). Recent evidence shows that mitomiRs contribute to inflammation and SAPS [106,107,108]. Overexpression of miR-210 in IMR90 cells induces a senescent phenotype, enhances DNA damage, and leads to oxidative stress by increasing ROS levels, possibly affecting ETC subunits I and II [82]. miR-21-5p and miR-203a-3p are involved in stress-induced senescence of HUVEC by reducing the level of the Drp1 protein, which activates mitochondrial fission [109]. 

In a study by Giuliani et al. [110], mitomiR-34a, -146, and -181a are overexpressed in replicatively senescent HUVECs, suppressing the activity of the apoptotic protein Bcl-2, which is responsible for the inhibition of apoptosis and autophagy. Initiated overexpression of the above SA-mitomiRs in young HUVECs leads to mitochondrial dysfunction and activation of proinflammatory caspase-1. The reduced functional capacity of Bcl-2 contributes to mitochondrial oxidative stress in neurodegenerative diseases [111]. Moreover, distinct sets of mitomiRs have been associated with Alzheimer’s, Parkinson’s, and Huntington’s diseases [112]. Another study by Giuliani et al. [113] further examined the role of SA-mitomiRs (let-7b, miR-1, miR-130a-3p, miR-133a, miR-146a-5p, miR-181c-5p, and miR-378-5p) in the oxidative, inflammatory, and energy status of senescent cells.

### 2.4. Epigenetic Changes

Over the last decade, a large number of studies have shown that epigenetic changes are one of the major causes of cellular senescence, affecting gene expression, but not disrupting DNA sequences. Epigenetic mechanisms include DNA methylation, histone modification, and changes in non-coding RNA expression [114].

DNA methylation occurs at CpG sites on cytosine residues via DNA methyltransferase enzymes (DNMTs) such as DNMT1, de novo DNMT3a, and DNMT3b [115]. Changes in the methylation profile are influenced by multiple factors. The methylation patterns in CpG sequences can be transmitted transgenerationally [116]. With aging, changes in methylation profiles are observed, which contribute to carcinogenesis, genomic instability [117], and an increased risk of age-associated diseases [118]. Moreover, hypomethylation is not a universal process that occurs in the genomes of all tissues and cells of an aging organism [119]. Age-related changes in DNA methylation are observed in specific regions of the genome; for example, methylation loss occurs in the 3’UTR, introns, and intergenic regions, but hypermethylation in CpG islands (CGI) and 5’UTR occurs in mice with age [120]. 

Inhibition of DNMT1 and DNMT3b initiates G1 cell cycle arrest and cellular senescence of human umbilical cord blood-derived multipotent stem cells (hUCB-MSCs) and increases p16^Ink4a^ and p21^CIP1^ levels [121]. Mice deprived of DNMT3a exhibit neuromuscular defects and a shortened lifespan [122]. The correlation of DNMT1 and DNMT3b expression with age was analyzed in a large-scale MARK-AGE study, where they determined a gradual decrease in DNMT1 expression up to 64 years of age and a linear decrease in DNTM3b expression with age [123]. 

Histone modification is another epigenetic mechanism of gene regulation through chromatin remodelling and includes acetylation, methylation, phosphorylation, ubiquitination, and sumoylation [116,117]. The most-studied modification is acetylation of lysine residues in the N-terminal tails of histones H3 and H4, which is performed by histone acetyltransferases (HATs) and is associated with transcription activation, and histone deacetylation mediated by deacetylases (HDACs), which leads to transcriptional repression [115,117]. 

The level of trimethylation of histone H4 by lysine 20 (H4K20me3) expression was observed to be higher, whereas the expression of acetylation of histone H4 by lysine 16 (H4K16ac) was substantially lower in senescent human diploid fibroblasts (HDFs) compared to young HDFs [124]. Mice with knockout of the *Zmpste24* gene (*Zmpste24*^−/−^), which is responsible for posttranslational processing and cleavage of prelamin A, exhibit premature aging and a phenotype characteristic of patients with rare hereditary laminopathies. *Zmpste24*-deficient mouse embryonic fibroblasts (MEFs) accumulated DNA damage and chromosome aberrations. They were highly sensitive to DNA-damaging agents and displayed early cellular senescence in culture. Reduced H4K16 acetylation in *Zmpste24*-deficient MEFs suggests that prelamin A accumulation impairs H4K16 acetylation. Knockdown of acetyltransferase MOF exacerbated the early cellular senescence phenotype of *Zmpste24*^−/−^ MEFs, indicating a role in maintaining H4K16 acetylation [125].

Histone methylation, particularly histone H3 trimethylation at lysine 4 (H3K4me3), lysine 36 (H3K36me3), and lysine 27 (H3K27me3), is significantly correlated with age and longevity [126]. NSD2 methyltransferase knockdown leads to increased mitochondrial mass, increased mitochondrial activation, and decreased levels of transcriptionally active H3K36me3 tag and ultimately initiates cellular senescence in human fibroblasts. Loss of NSD2 leads to a decrease in genes associated with the cell cycle and promotes senescence, in part, by activating the RB-mediated pathway [127]. In patients with severe Alzheimer’s disease, there is a decrease in the expression of active H3K4me3 tags and an increase in the level of repressive H3K27me3 tags, which confirms the role of epigenetic mechanisms in the development of neurodegenerative diseases [128]. 

## 3. Senescence-Associated Secretory Phenotype (SASP)

Despite irreversible cell cycle arrest, aging cells remain metabolically active [129], secreting a multitude of factors into the surrounding microenvironment, thereby mediating the “bystander effect” (BE), which promotes the accumulation of senescent cells in tissues [130] and initiates the senescence-associated secretory phenotype (SASP) [131]. SASP is associated with the production of a wide range of molecules, including pro-inflammatory mediators, such as interleukins and chemokines, growth factors, proteases, and a variety of other proteins and non-protein molecules [132]. 

Another factor in remodelling the microenvironment through intercellular communication in the SASP is extracellular vesicles (EVs). They transport SASP factors, including miRNAs (sEV-miRNAs), which modulate the phenotype of recipient cells [131]. The uptake of such exosomes by bystander cells can potentially lead to genomic instability, metabolic reprogramming, and metastasis [133]. Primary human dermal fibroblasts exhibit a four-fold increase in the secretion of small EVs during premature stress-induced cellular senescence. Simultaneously, analysis of miRNAs in sEVs allowed us to distinguish senescent cells from quiescent cells [134]. 

Many diverse signalling cascades are involved in the regulation of SASP [135]. We will focus on the role of the transcription factor NF-kB as the main inducer of the SASP.

### 3.1. Transcription Factor NF-kB

Nuclear factor-kappa B (NF-κB) is a family of proteins involved in the body’s response to stress, controlling the expression of hundreds of genes that regulate significant physiological processes such as immunity, inflammation, proliferation, and cell death [136]. The NF-κB family consists of five related proteins: p50 (NF-κB1), p52 (NF-κB2), p65 (RelA), RelB, and c-Rel (Rel) [137]. 

In resting cells, without a stimulus, NF-kB is sequestered in the cytoplasm through interaction with inhibitor proteins, particularly IκBα [138]. Activation of NF-kB is triggered by an initiating signal from various agents, such as inflammatory molecules such as TNF-α [137]. This leads to the activation of the IKK (IκB) complex, which consists of two catalytic subunits, IKK1 (IKKα) and IKK2 (IKKβ) kinases, and one regulatory subunit represented by the essential modulator of NF-κB (NEMO or IKKγ) [139]. Activation of NF-κB and triggering of IKK leads to phosphorylation of the IκBα inhibitor and its subsequent polyubiquitination and degradation by the proteasome. This allows NF-kB to penetrate the nucleus and control the transcription of its target genes [138]. 

One of the key factors that provokes NF-kB is DNA damage [140]. As mentioned earlier, failure to repair the damage results in the formation of a persistent DDR response characterized by cell cycle arrest, prolonged activation of ATR/ATM kinases, and SASP secretion through various pathways including NF-kB, ultimately leading to cellular senescence [141]. There is evidence reporting that ATM is a key activator of sustained NF-kB during DNA damage [142]. This is supported by a study by Zhao et al. [143], where *Ercc1^−/^^Δ^* mice with an impaired DNA repair system were used as an animal model of progeroid syndrome. *Ercc1^−/^^Δ^* mice and aging wild-type (WT) mice exhibit elevated ATM levels accompanied by NF-kB hyperactivation. Inhibition of *ATM* in *Ercc1^−/^^Δ^* mice suppresses the NEMO-mediated activation of NF-kB, reduces the expression of SASP markers, and slows down the processes of aging induced by oxidative stress. Simultaneously, mice heterozygous for *ATM* (*Ercc1^−/^^Δ^Atm*^+/−^) also showed a decrease in NF-kB activity and displayed an age-related phenotype. Thus, ATM may serve as a therapeutic target for controlling the NF-kB and SASP families to slow down the aging processes in both natural and induced cellular senescence.

Exposure of senescent fibroblasts to antioxidants decreases ROS levels and completely inhibits NF-kB activation, indicating the role of mitochondrial dysfunction as a factor stimulating NF-kB in aging. Meanwhile, the direct inhibition of NF-kB had no effect on the amount of ROS in senescent cells. Co-culture of young fibroblasts with senescent fibroblasts led to an increase in the level of ROS and enhancement of DDR in control proliferating MRC-5 cells, demonstrating the bystander effect. Repeated co-culture, but using antioxidants or specific NF-kB inhibitors, reduced BE in all senescent cell lines studied, but with different time intervals of manifestation [144]. The exposure of mouse embryonic fibroblasts (MEF) to laser irradiation initiates the production of mtROS, which activates NF-kB. At the same time, the use of mitochondrial inhibitors enhances NF-kB activation, whereas incubation with antioxidants eliminates it. Thus, laser-induced activation of NF-kB is mediated by ROS generation [145]. 

### 3.2. Cytokines

Long-term inflammation is often linked with natural and stress-induced cellular senescence, and it can be triggered by a variety of factors. Chronic inflammation can result in DNA damage and oxidative stress [146]. Immune cells have a critical function in identifying and eliminating senescent cells, and they too are influenced by SASP, causing immunosenescence. Chronic inflammation has a detrimental effect on the immune system by accelerating the senescence of immune cells, which impairs their ability to eliminate senescent cells and inflammatory molecules [147]. This leads to a vicious cycle of inflammation and senescence, characterized by an inability to effectively combat inflammation and an increased risk of age-related diseases. With age, senescent cells accumulate, creating a proinflammatory environment, secreting SASP factors, and affecting neighboring cells [148], thus becoming a source of chronic inflammation. However, the role of some SASP components is twofold; on the one hand, they have immunomodulatory effects, contributing to the destruction of damaged and senescent cells, but on the other hand, their uncontrolled expression may contribute to cancer progression, chronic inflammation, and a number of other pathologies [149]. 

Cytokines are small secreted proteins that lead to the activation of immune cells [150] and are directly produced by activated immune cells [151]. Altered cytokine expression is associated with a tendency to exhibit a pro-inflammatory phenotype underlying age-related diseases, which has been provisionally termed “inflammatory aging” [152]. 

The NF-κB signalling pathway mediates a number of important cellular functions, including the regulation of immune and inflammatory responses. One mechanism of inflammation activation is through Toll-like receptor 9 (TLR9) [153] via NF-kB, which induces the expression of some pro-inflammatory cytokine genes [154]. TLR9 can recognize and bind to cf-mtDNA, leading to the activation of the immune system [155,156]. In elderly people, cf-mtDNA levels are positively correlated with inflammatory markers, including C-reactive protein, sTNF-α, sTNFR1, and IL-6 [68]. Chronic inflammation induced by NF-κB1 knockout accelerates senescence and causes premature aging in mice, as determined by increased levels of cytokines, particularly IL-6, reduced tissue regeneration, and telomere dysfunction [157]. IL-6 and IL-8 are known to be under the control of NF-kB, which marks the role of NF-kB as one of the transcription factors that control proinflammatory SASP [144].

Mitochondria are involved in the modulation of immune responses. Oxidative stress arising during mitochondrial dysfunction may be another cause of chronic inflammation [158]. Oxidative stress activates the secretion of pro-inflammatory cytokines and SASP, including TNF-α, IL-1β, IL-6, and IL-8 [159]. In particular, ROS act as signalling molecules that induce cytokine activation through various pathways [160]. Persistent DDR correlates with IL-6 production, and ATM depletion reduces IL-6 levels by 70%, confirming the role of DDR in stimulating inflammatory cytokine secretion [161]. Wu et al. [141] reported an inseparable link between DDR and cytomegalovirus (HCMV)-induced SASP production and cellular senescence. Prolonged exposure to latent infection with HCMV leads to T-cell terminal differentiation, resulting in the accumulation of exhausted CD28 T-cells that secrete TNF-α and IFN-γ, promoting inflammatory senescence. During productive infection, infected cells exhibit senescent phenotypes, such as cell cycle arrest and SASP.

miRNAs are of particular importance in inflammatory processes. miR-21 regulates inflammation by influencing the NF-κB/NLRP3 pathway [162]. Overexpression of miR-21 in macrophages leads to increased expression of IL-1β, TNF-α, IL-6, and IL-8, initiating a pro-inflammatory phenotype [163]. In contrast, another study highlighted miR-21 as a negative feedback factor in the inflammation process, fulfilling an anti-inflammatory function in chronic rhinosinusitis with nasal polyps (CRSwNP) [164]. miR-146a/b is a negative regulator of inflammatory cytokine expression. Overexpression of miR-146a/b suppresses the aging-related secretion of IL-6 and IL-8, but an increase in miR-146a/b has been observed in aging fibroblasts that secrete high levels of inflammatory cytokines [165]. A multitude of exosomal miRNAs are involved in inflammatory regulation and exert both proinflammatory and anti-inflammatory effects [166].

## 4. Radiation-Induced Cellular Senescence

A considerable amount of experimental data indicates that ionizing radiation (IR) can lead to accelerated aging processes under the influence of various mechanisms [167]. Most of these biological effects are stimulated by IR-mediated DNA damage, mitochondrial dysfunction leading to excessive ROS production and oxidative stress, and changes in miRNA expression and epigenetic profiles.

Radiation has a significant effect on telomeres. Radiation therapy impairs the structural and functional integrity of telomeres in normal human cells, which appears to be one of the reasons for the shortened lifespan [168]. 

To determine the sensitivity of telomeres to radiation-induced DNA damage and activation of the DDR system, human MRC5 fibroblasts and mouse embryonic fibroblasts (MEFs) were exposed to X-rays at different cumulative doses. An increase in the number of γH2AX and telomere-associated foci (TAF) and an increase in SA-β-gal activity were observed [29]. Thus, telomeres are potential targets of genotoxic stress, particularly IR-associated stress, which may have significant consequences for cellular senescence.

### 4.1. DNA Damage

DNA damage, particularly DSBs, is mediated not only by endogenous agents, such as ROS [39], but also by genotoxic environmental factors, including ionizing radiation [3,169]. IR-induced DSBs can occur in irradiated cells due to the direct effect of radiation or indirectly through water radiolysis and generation of ROS [3,170]. The direct IR pathway involves collision of a high-energy photon with a DNA strand, which causes the phosphodiester backbone to break [171]. The indirect effect of IR is mediated by increased formation of ROS, which is usually caused by water radiolysis. Absorption of high-energy γ-ray and X-ray photons causes the excitation and ionization of water molecules, leading to the production of free radicals and electrons that damage DNA [172]. 

It has been widely suggested that radiation therapy may accelerate cellular senescence, leading to premature aging and age-associated diseases. Intracellular changes caused by anticancer treatments, such as radiotherapy, can lead to premature aging by altering DNA structure, gene expression, mutations mediated by transcription regulator cascades, protein expression, and increasing damage from ROS [2,173,174]. Thus, individuals undergoing radiation therapy develop age-related diseases and phenotypes earlier than members of the general population, which is probably due to tissue damage that initiates disruption of DNA structural integrity, thereby activating the DDR system [4] and accelerating aging processes [175]. Radiosensitivity increases with age [13] and is determined by the extent of radiation damage to DNA and the individual organism’s ability to successfully repair this damage [176]. In an animal model, female rats of postpubertal age (7 weeks) showed greater sensitivity to the mutagenic effect of radiation than infant (1 week) and prepubertal (3 weeks) females [177]. 

The previously mentioned phosphorylated histone γH2AX is a specific biomarker of the cellular response to DNA damage, primarily to DSBs [30]. Ulyanenko et al. [178] studied the formation of DSB markers (including γH2AX) in MSCs exposed to chronic γ-irradiation (0.1 mGy/min) and acute γ-irradiation (30 mGy/min) at different cumulative doses. Significant dose-effect differences were found between chronic and acute irradiation, in which in the latter case IR resulted in a pronounced linear dose-dependent increase in the number of γH2AX foci. Low-dose irradiation demonstrated a nonlinear pattern. In another study, low dose rates of chronic IR were found to inhibit cell proliferation. Chronic γ-radiation of primary keratinocytes and fibroblasts at dose rates ranging from 6 mGy/h to 20 mGy/h for 7 days induced the formation of low numbers of DSBs. Conflicting changes in the levels of histone H2AX have been observed after low-dose chronic irradiation, with a decrease being noted [179].

Most DNA damage caused by radiation is quickly fixed, but some lesions remain in the cell nucleus for several days to months (Table 2). These lesions, including unrepairable DSBs, pose a significant threat to organisms and can lead to persistent cell and tissue dysfunction, as well as delayed effects of radiation, such as cell death, mutation, senescence, or carcinogenesis [180]. Exposure to radiation at an early age may result in the formation of γH2AX foci or persistent DNA damage foci (PDDF), which may be linked to the aging process of the brain and may cause a shorter lifespan for irradiated animals [181]. According to the study of Vaurijoux et al. [182], depending on the dose (1 or 5 Gy), up to 5–10% of ionizing radiation-induced foci (IRIF) persist in 10–70% of HUVECs 24 h after irradiation, they observed persistent IRIFs for 7 days after irradiation, and more than 70% of irradiated cells (5 Gy) had at least one persistent IRIF 24 h later. 

Regardless of the inducing stimulus, senescent cells share common mechanisms for activation and maintenance of senescence, which are associated with p53, p21, p16, and pRB proteins [2]. Li et al. [184] observed increased expression of p-p38, p38, and p16^INK4a^ proteins in the spleen of irradiated mice. Simultaneously, IR decreases the level of SIRT1, which represses the transcriptional activity of p53 by deacetylation [185], resulting in increased expression of p53 and p21^CIP1^. In general, radiation-induced cellular senescence is associated with DNA damage and genomic instability.

### 4.2. Mitochondrial Dysfunction

The mitochondria play an important role in radiation-induced cellular senescence. Radiation leads to mitochondrial dysfunction, impaired antioxidant activity and mtDNA structural integrity, excess ROS, oxidative stress, and apoptosis [186,187]. In response to IR, quiescent microvascular HMVEC-L activates senescence through two independent mechanisms: the mitochondrial dysfunction associated with oxidative stress and the p53 pathway [188]. In human lung carcinoma A549 cells, X-ray irradiation increased the mtROS level and enhanced ETC function [189]. Rat bone marrow mesenchymal stem cells (rBMSCs) show an increase in mtROS and altered expression of miRNAs after high-dose X-ray irradiation [190]. In general, ROS are the main source of IR-induced free radical cell damage [167]. Late post-radiation effects are primarily caused by chronic oxidative stress, making it a significant adverse effect of IR exposure and a key target for radiological protection [191].

The effect of radiation exposure on NAD+ levels is poorly understood and, at some points, there are conflicting data. The expression of SIRT1 and NAMPT decreases 5 days after IR, with irradiation leading to decreased levels of phosphorylated AMPK in parotid gland tissues and decreased NAD+. Using AMPK activators resulted in a reduction in compensatory proliferation on days 6, 7, and 30 following IR, and it also reversed the chronic salivary gland dysfunction on day 30 that occurred after IR [192]. In a study by Liao et al. [193], IR caused *NAMPT* activation 15–30 min after irradiation in human oral keratinocytes (HOKs) and HUVECs, which was probably a rapid stress response, and reduced nuclear NAD levels by 40–50%. However, the authors did not examine the changes in *NAMPT* expression several days after radiation exposure. It is also reported that AMPK-mediated phosphorylation of NAMPT S314 significantly restores NAD+ levels in irradiated cells, promotes DNA repair, and enhances cell viability under IR conditions.

Mutations in mtDNA lead to mitochondrial diseases and are involved in the development of progeroid syndromes [194]. Prithivirajsingh et al. [195] observed significant levels of Δ-mtDNA^4977^ deletion 72 h after irradiation in normal human fibroblast cell lines, but did not reveal a dose-response relationship. Both the AT and ATSV40 lines showed increased accumulation of mtDNA deletions after radiation, indicating that IR induces an increase in mtDNA deletions. In summary, this increase in mtDNA deletions is radiation dose-independent, requires 72 h to accumulate to detectable levels, occurs in both normal and tumor cell lines, varies in magnitude, and shows no correlation with radiosensitivity. Such point mutations and deletions in mtDNA accumulate as a result of IR, leading to impaired mitochondrial function and the subsequent release of ROS.

In another study [196], the brain and spleen tissues of male mice that were exposed to radiation (2 and 5 Gy) were evaluated for the presence of mtDNA deletions 1 and 4 months after exposure. Four months later, the level of mtDNA deletion in tissues from mice exposed to 5 Gy was higher than that in animals exposed to 2 Gy and controls. In the temporal aspect, it was demonstrated that the number of mtDNA deletions after 4 months was higher than 1 month after X-ray in both experimental groups. This emphasizes the dependence of the mtDNA deletion level on the age of the animal, radiation dose, tissue type, and duration of the post-radiation period.

After IR in human glioma LN229 cells with silencing of *SIRT3* expression, there was worsened oxidative damage to mtDNA, as measured by the accumulation of 8-oxoG and Δ-mtDNA^4977^ deletion, resulting in more severe mitochondrial dysfunction and increased apoptosis compared to cells without *SIRT3* inhibition [197]. This confirms early data that SIRT3 dysfunction is associated with impaired repair and cellular metabolism, which aggravates IR-induced mtDNA damage, disrupts mitochondrial integrity, and leads to mitochondrial genome instability. 

Schilling-Tóth et al. [198] found that the long-term effects of IR exposure on immortalized radiation-sensitive (S1-hTERT) and normal (F11-hTERT) fibroblasts persisted for up to 63 days. In F11-hTERT cells, the common deletion (CD) levels increased until 35 days post-IR and then returned to control levels by day 49. In S1-hTERT cells, the CD level normalized by day 42, but a second wave of CD incidence appeared by day 49 and persisted until day 63 post-IR, indicating radiation-induced instability in the mitochondrial genome.

The use of cf-mtDNA as a biomarker for radiation exposure is of considerable interest. There are data on the appearance of cf-mtDNA in the urine of rats after irradiation, but before IR, they were absent [199]. Bisserier et al. [200] measured cf-mtDNA levels in the blood plasma of astronauts 10 days before launch, on the day of landing, and 3 days after return and found a significant increase in cf-mtDNA in two cases: on the day of landing and a few days later. Gene expression analysis of peripheral blood mononuclear cells (PBMC) revealed increased markers of oxidative stress, DNA damage, and inflammation. The authors suggested that excess cf-mtDNA may be a biomarker of radiation-induced stress during spaceflight. We have also demonstrated that radon, a radioactive gas, increases the level of cf-mtDNA both in patients with radon-induced lung cancer and in individuals living in areas with high radon levels, which confirms the role of cf-mtDNA as a promising biomarker of radiation exposure [64]. 

Radiation-induced cellular senescence manifests as mitochondrial dysfunction in young MRC5 bystander cells, increasing the level of ROS, and during 6 days of co-culture with inducer fibroblasts, there is activation of the DDR [144]. BE triggered by mitochondria affects not only ROS but also the release of mtDNA in exosome-like vesicles [201].

The mechanism of the interaction between IR and mitophagy is not completely clear, but it is actively discussed in terms of the effect of irradiation on cancer cells. It has been reported that mitophagy induced by mtROS can enhance the radiosensitization of HeLa cells [202]. Moreover, the effect of X-rays on A549 and Calu-3 cells increases mitophagy to a peak level 8 h after irradiation [203]. Another study showed that IR damages mitochondria by deforming them and initiates mitophagy in both PANC-1 and SW1990 cells by increasing the levels of the mitophagic sensors Parkin and BNIP3. After a period of 48 h following X-ray irradiation, it was observed that there was a rise in the spatial proximity between the lysosomes and mitochondria in both PANC-1 and SW199 cells [204]. The effect of high-dose irradiation leads to a decrease in the mitochondrial membrane potential, which correlates with defective protein import. Recovery of membrane potential, but not of import, was observed during a 12 h period after IR exposure in AG1522 fibroblasts [205]. 

Meng et al. [206] demonstrated that low-dose-rate IR causes the accumulation of ROS, leading to oxidative stress in human normal 48BR cells. Moreover, low-dose-rate IR induces modifications in mitochondrial morphology and mitochondrial membrane potential, which are not observed in cells subjected to high-dose-rate IR or non-irradiated cells. On the other hand, high-dose-rate IR increased PINK1 levels and exhibited a slight increase in Parkin levels, which did not occur with low-dose-rate IR. These results indicate that a high dose rate induced rapid autophagy-dependent degradation of mitochondria via PINK1, whereas a low dose rate did not result in significant mitophagy. In contrast, low-dose-rate IR causes repression of mitophagy and an accumulation of damaged mitochondria, which then leak excess ROS and trigger oxidative stress responses.

Local heart irradiation induces long-term changes in cardiac mitochondrial membrane function, including a significant increase in the expression of Bax at time points from 6 h to 2 weeks and the Bax/Bcl2 ratio from 6 h to 6 months after IR, as well as a reduction in mitochondrial membrane potential. Additionally, there was an increase in PINK1 at 2 weeks and a decrease at 10 weeks after irradiation, while the levels of Parkin remained unchanged [207].

Chronic irradiation caused changes in the heart proteome, with the number of downregulated mitochondrial and structural proteins increasing in a dose-dependent manner, indicating an increase in mitochondrial dysfunction [208].

The change in 8-OHdG levels in response to IR provides mixed results. Healthcare workers occupationally exposed to IR showed a quantitative increase in superoxide (O_2_•^−^) levels, but analyses of 8-OHdG levels did not show statistical significance. No correlation was found between 8-OHdG levels and O_2_•^−^ levels [209]. In contrast, another study on the relationship between occupational exposure and oxidative damage in interventional physicians demonstrated an increase in 8-OHdG levels in an experimental group [210]. In addition, increased expression of 8-OHdG has been found in astronauts after flight [200]. 

### 4.3. Associated miRNAs

Increased attention to miRNAs in radiation biology is associated with the search for molecular diagnostic biomarkers to assess the level of IR-induced damage to organisms, including radiation-related premature cellular senescence (Table 3) [211]. 

Nikiforova et al. [213] identified 30 miRNAs whose levels changed after irradiation of thyroid cells without showing a dose-dependent curve (1 and 10 Gy). Four patterns were observed after IR exposure: (i) miRNA expression level decreased at 4 h, then recovered at 24 h; (ii) miRNA expression increased at 4 h and then returned to normal at 24 h; (iii) miRNA expression decreased; or (iv) miRNA expression increased at both time points.

miR-34a overexpression enhances premature IR-induced cellular senescence by targeting the *c-Myc* oncogene. The human non-small-cell lung cancer (NSCLC) cell lines A549 and H460 exhibited a dose-dependent increase in SA-β-gal levels in response to IR. Analysis of miR-34a showed increased expression in correlation with the radiation dose in both A549 and H460 cells. Ectopic overexpression of miR-34a enhances IR-induced senescence, whereas miR-34a knockdown attenuates it. Time course studies showed that miR-34a expression levels increased in a time-dependent manner in irradiated A549 and H460 cells. Peak levels were reached 3 days after IR exposure and remained high even after 5 days. This persistent upregulation of miR-34a in NSCLC cells after IR exposure correlates with IR-induced senescence, suggesting a role for miR-34a in modulating senescence in lung cancer cells [212].

Summerer et al. [225] identified eight miRNAs, including miR-21-5p, whose levels differed significantly in plasma from patients with head and neck squamous cell cancer (HNSCC) after radiation therapy compared to samples from healthy donors. 

The expression of miR-21 was increased in AG1522 cells that were subjected to acute or chronic irradiation of 10 cGy or acute irradiation of 400 cGy [226]. Another study found that radiation promotes the expression of miR-21 in brain tissue, and notably, miR-21 levels continue to rise steadily for 1 year following radiation exposure [227].

When mice were irradiated with γ-rays and Fe^−56^ ions, the miRNA signatures showed a dependence on the type of radiation and radiation dose. For example, 24 h after γ-irradiation, six differentially expressed miRNAs, including miR-146a, were detected in the blood of irradiated mice [223]. 

Analysis of postmortem samples from the cardiac left ventricle taken from workers at the Mayak plutonium enrichment plant revealed significantly increased expression of miR-21 and miR-146a in the group with the highest exposure dose compared with the control and lower-dose groups [208]. A substantial increase in miR-21 expression was observed in irradiated rat hearts at all evaluated time periods, including 2 days (30%), 9 days (26%), and 45 days (35%). Additionally, miR-15b expression was significantly downregulated by 31%, while miR-1 expression was also downregulated by 35% compared to non-irradiated rats after 45 days [228]. 

Gao et al. [229] discovered an increase in the expression of four miRNAs, specifically miR-144-5p, miR-144-3p, miR-142-5p, and miR-19a-3p, in rat blood samples 2 weeks after exposure to radiation.

EVs play an important role in the manifestation of the effects of radiation on cells. In particular, exosomes released by irradiated cells into the culture media demonstrate the so-called “radiation-induced bystander effect” (RIBE) in recipient cells through intercellular communication, which is manifested by the production of many factors including inflammatory mediators, ROS, and various miRNAs [230]. The role of miRNAs in RIBE has recently been extensively investigated; however, the underlying mechanisms have not been fully elucidated.

RIBE in MRC-5 cells demonstrated an altered expression profile of miRNAs. A total of 136 miRNAs were identified in both irradiated and non-irradiated cells, of which 67 were downregulated and 69 were upregulated. miR-21 is the most upregulated miRNA, with more than a two-fold increase in expression after irradiation [231]. 

Human dermal fibroblasts exhibit a senescent phenotype two weeks after IR exposure, as demonstrated by changes in cell morphology, increased SA-β-gal activity, overexpression of SASP genes, and increased levels of miR-29a-3p, miR-30a-3p, miR-34a-5p, miR-24a-3p, and miR-186-5p in EVs isolated from the media [105]. 

Dinh et al. [232] found differences in the levels of miR-29a-3p and miR-150-5p in exosomes purified from conditioned media of irradiated NCI-H460, A549, and NCI-H1299 cells and this difference persisted with each fraction of radiation for up to 3 days of treatment. Similarly, exosomal miR-29a and miR-150 were also lower in irradiated MRC5 and IMR90 cells. However, radiation increased the intracellular expression of miR-29a and miR-150 in tested NSCLC cell lines (NCI-H460, A549, and NCI-H1299) and in MRC5 and IMR90 as early as 2 h after irradiation. This increase persisted with each fraction of radiation for up to 3 days of treatment. Such a decrease in the blood levels of miR-29a and miR-150 may be a regulated process rather than a reflection of the decreased expression of miRNAs in cells.

In a previous review, we discussed the extensive role of mitochondrial miRNAs in radon-induced lung cancer development, showing that prolonged radon exposure led to increased expression of miR-34a, miR-15a-5p, miR-19a-3p, and miR-21-5p and decreased levels of miR-144-5p, miR-193b-5p, and miR-32-3p [233].

### 4.4. DNA Methylation and Histone Modification

Epigenetic mechanisms are sensitive to environmental factors, including the effects of ionizing radiation. For example, an increase in the number of hypermethylated spermatozoa has been observed in healthcare workers exposed to occupational IR [234]. Moreover, similar to target-cell irradiation, non-targeted radiation exposure to neighboring cells also leads to changes in the epigenetic profile, exhibiting a transgenerational effect [235]. In a study of irradiated zebrafish offspring (F0), the formation of a large number of differentially methylated regions (DMRs) was detected in F1 embryos. Five DMRs showed persistent changes in DNA methylation up to the F3 generation embryos [236]. Increased intramitochondrial ROS leads to mtDNA damage, causing global DNA hypomethylation via decreased DNMT activity and transmission of these changes to the progeny of irradiated cells [191].

Many studies have demonstrated that irradiation initiates DSBs, DNA hypomethylation, and cellular senescence [237]; increases DNMT1 levels; and decreases DNMT3a and DNMT3b expression [238] in bystander tissues. DNMT1 and DNMT3a play a crucial role in maintaining the memory of murine embryonic stem (ES) cells that have been exposed to IR. This memory is evident in the conditioned media from the progeny of exposed cells inducing DNA damage and homologous recombination in bystander cells. It has been observed that ES cells exposed to γ-radiation retain the memory of the insult for several weeks. By inhibiting the *DNMT1* and *DNMT3a* genes in ES cells, it is possible to prevent the transmission of genomic instability to bystander cells [239]. 

A week after radiation exposure, slight increases in the expression of DNMT1 and DNMT3a were detected in the cardiac tissue of irradiated mice. However, at the 90-day time point, a notable decrease in DNMT3a and DNMT3b levels was observed in mice exposed to protons and Fe^56^ [240].

Fractionated low-dose radiation exposure (0.5 Gy) for 10 consecutive days led to the accumulation of γH2AX foci in thymus cells, which supports the notion that such exposure has a significant impact on the balance of DNA repair systems within cells. Additionally, fractionated exposure resulted in reduced global DNA methylation, which was accompanied by decreased levels of DNMT1, DNMT3a, and DNMT3b. Moreover, the level of H4K20me3 in thymus tissue from female mice exposed to fractionated low-dose radiation was lower than in the control group [241].

Wang et al. [242] demonstrated that mice subjected to chronic exposure for 10 days experienced a loss of global DNA methylation. Moreover, these mice exhibited lower levels of DNMT1 mRNA and protein in their peripheral blood mononuclear cells (PBMC), kidney, and liver tissues.

The histone acetyltransferase MOF, through the acetylation of H4K16, plays a crucial role in DDR and DSB repair. Reduced H4K16ac activity correlates with aberrant DDR in response to IR, whereas *MOF* inactivation leads to loss of H4K16ac and impaired proliferation of mammalian cells (HEK293, MCF7, HCT116, GM5849, and HL60 cells) [243]. *MOF* mutations and knockdown reduced the survival of *Drosophila melanogaster* and SL-2 cells after irradiation, and mutant embryos exhibited an increased sensitivity to radiation. In contrast, an increase in H4K16ac levels in SL-2 cells was observed after γ-ray treatment [244]. Another study showed that IR causes a decrease in H4K16ac levels and an increase in H2AX phosphorylation in cells (HeLa, HeLa EM2-11-TPX2, and MCF7), dependent on the depletion of the TPX2 protein involved in the cell cycle [245]. 

The histone demethylase KDM5B, which is involved in H3K4me3 demethylation, is recruited to DSB sites and participates in DNA repair after genotoxic damage. KDM5B knockdown in HEK293 cells showed persistence of radiation-induced γH2AX foci, p53 hyperactivation, cell cycle delay, and a significant decrease in cell viability [246]. Other studies have reported the accumulation of the repressive H3K27me3 tag and loss of H3K4me3 at injury sites after IR [247]. H3K27 methylation has been observed at DSB sites and after oxidative damage [248]. 

Rath et al. [249] reported a radiation-induced decrease in H3K27me3 in tumor cell lines (U251, MDA-MB-231, and A549) occurring with the participation of demethylase UTX. In contrast to the results obtained in tumor cells, IR did not affect H3K27me3 levels in normal human fibroblast cell lines (MRC5 and MRC9). Treatment of tumor cells prior to irradiation with the H3K27 demethylase inhibitor GSKJ4 prevents a radiation-induced reduction in H3K27me3 and increases the radiosensitivity of tumor cells but not normal cells.

The demethylase KDM2A involved in H3K36me2 demethylation is recruited to DSB sites through its demethylase activity and zinc finger domain and modulates ubiquitination and recruitment of the 53BP1 (p53-binding protein 1) protein to lesion sites. The depletion of KDM2A or destruction of its zinc finger domain initiates the accumulation of micronuclei after IR. Moreover, irradiated KDM2A-deficient cells exhibit premature exit from the G2/M checkpoint, resulting in early entry into mitosis without the proper repair of DSBs [250]. 

## 5. Ionizing Radiation and SASP

Radiation-induced cellular senescence mediates the secretion of soluble (sSASP) and exosome-packed (eSASP) SASP factors, exhibiting different quantitative and qualitative compositions [251]. 

IR through a bystander effect exhibits off-target effects on unexposed cells through intercellular communication or through secreted signals present in the media of directly exposed cultures and in exosomes extracted from these media [252]. Al-Mayah et al. [253] demonstrated that treatment of non-IR-exposed cells with irradiated MCF7 cell conditioned media (ICCM) induces chromosomal aberrations and DNA damage. Moreover, the use of RNase suppressed BE in ICCM-treated cells, reducing the level of chromosomal mutations, which was not observed when irradiated cells were treated directly. The treatment of normal cells with exosomes extracted from ICCM also increases the level of damage, and the action of RNase reduces this effect.

The importance of exosomes in radiation-induced BE is supported by the exposure of unirradiated cells to ICCM from which exosomes and other sEVs have been removed, resulting in no damaging effects [254]. These data suggest the role of exosomal RNAs, in particular miRNAs, as key mediators of radiation-induced BE and, consequently, SASP.

### 5.1. Role of IR in the Induction of NF-kB

The exact mechanisms by which NF-kB is activated by non-inflammatory stimuli such as IR-associated genotoxic stress is not yet fully understood. Early evidence suggests a p53-dependent inactivation of NF-kB following irradiation [255]. DSBs signals initiate DDR via ATM, sending information to p53 and NF-kB, which determine cell fate [256]. Radiation-induced DSBs lead to rapid activation of NF-kB via NEMO, increasing p65 protein expression and suppressing IκB-α inhibitor protein expression [257]. P53-induced protein with a death domain (PIDD) is regulated by p53 and is induced in response to DNA damage while activating caspases or NF-kB translocation [258]. Depletion of PIDD results in impaired activation of the NF-κB response to genotoxic stress by inhibiting sumoylation, phosphorylation, and ubiquitination of NEMO [259]. According to other data, atypical activation of NF-kB induced by ionizing radiation is not affected by the p53 status [260].

Li et al. [261] determined that IR and short-wavelength ultraviolet irradiation (UV) have two different mechanisms of NF-kB induction. Similar to the effect of TNF-α, the action of γ-radiation on HeLa and 293 cells leads to p65 translocation and increased NF-kB binding activity. Radiation initiates partial and slow degradation of IκB-α, dependent on Ser-32 and Ser-36 phosphorylation, and activates IKK for the nuclear translocation of NF-kB. 

Examination of the NF-kB activation pathway in ataxia telangiectasia (AT) cell mutants of the *ATM* gene showed that after irradiation, AT cells do not degrade IκB-α and exhibit reduced NF-kB binding activity; however, *ATM* WT overexpression restores radiation-induced degradation of IκB-α. In contrast, in healthy irradiated cells, within the first two hours after IR, IκBα was actively degraded by the proteasome, resulting in translocation of p65 to the nucleus and an increase in NF-kB binding activity [262]. 

According to the available data, long-term radiotherapy can trigger NFκB activation, leading to heightened production of pro-inflammatory cytokines in the intestines, which ultimately results in more extensive tissue damage [263]. Irradiation selectively activated NF-kB in the spleen, mesenteric lymph nodes, and bone marrow of irradiated mice, mediating an increase in the mRNA levels of TNF-α, IL-1α, IL-1β, and IL-6. Simultaneously, inhibition of the *p50* gene attenuates IR-induced activation of NF-kB, significantly reducing cytokine mRNA expression [264]. Examination of splenocytes derived from 56-week-old *p53*+/− mice irradiated at an early age (8 weeks) and at a later age (17, 30, and 41 weeks) shows increased NF-kB activation and enhanced IL-6 production in mice exposed to radiation at a young age. Thus, *p53*+/− mice irradiated at 8 weeks of age showed strong phosphorylation of NF-kB mediators compared to controls, whereas *p53*+/− mice irradiated at 17, 30, and 41 weeks of age showed no change in p65 phosphorylation [265]. 

Nelson et al. [144] found that in all modes of fibroblast senescence, there was an increased secretion of IL-6 and IL-8. Inhibiting NF-κB was effective for both interleukins in IR-induced senescence but was slightly less effective for MRC5 than for IMR90. High ROS levels in radiation-induced senescence stimulate NF-κB activation; however, suppression of NF-κB has no effect on senescence-associated mitochondrial dysfunction (SAMD).

### 5.2. TNF-α, IL-1α, IL-1β, IL-6, and IL-8

The effect of IR on inflammatory processes is currently being investigated. Endothelial cells (ECs) are extensively used as a cellular model for research because they play an important role in inflammation. Radiation exposure is known to shift ECs to a pro-inflammatory state [8]. Irradiation of ECs leads to dose-dependent G1 cell cycle arrest, overproduction of IL-6 and CCL2, and increased β-Gal activity and IGFBP7 secretion, indicating premature senescence [266]. Another study showed that γ-irradiation of HUVECs enhanced IL-6 and IL-8 production [267]. The endothelium, which is a major target of radiation damage, contributes significantly to the development of cardiac damage. Radiation-induced endothelial dysfunction in mice exposed to IR after 16 weeks is characterized by impaired energy metabolism, premature endothelial senescence, and increased oxidative stress and inflammation, which are major contributors to long-term vascular dysfunction [268].

Microglia are activated by IR, leading to cytokine release, inflammation, activation of the DDR system, and metabolic changes, thereby contributing to radiation-induced brain aging and an increased risk of neurodegenerative diseases [4]. Irradiation of corilagin-pretreated BV-2 microglial cells demonstrated decreased expression of cytokines IL-1β, TNF-α, and IL-6 compared to untreated irradiated cells [257]. 

NF-κB has been reported to regulate the production of a wide range of inflammatory mediators after IR exposure, with some cytokines having strong pro-inflammatory effects that may contribute to increased damage after exposure, while others induce tissue regeneration [269]. Thus, activation of NF-kB in the organs of irradiated mice increases the mRNA levels of TNF-α, IL-1α, IL-1β, and IL-6 [264]. BV-2 treatment with corilagin suppressed radiation-induced activation of the NF-κB pathway [257]. Moreover, atypical radiation-induced NF-κB activation has slower kinetics, but is regulated by the same subsets of NF-κB-dependent genes as the canonical cytokine-stimulated NF-κB pathway [270].

TNF-α increased by 20% in the irradiated group of rats 2 days after irradiation and remained elevated 9 days later. Forty-five days after irradiation, the levels of TNF-α in irradiated rats were found to be significantly higher than those in non-irradiated rats, with an increase of over 14% [228]. TNF-α and IL-1α mRNA levels were increased in fibrosis-sensitive (C57BL/6) mice after irradiation, with TNF-α levels peaking at days 1 and 7 after 5 Gy and IL-1α levels peaking at days 56, 112, and 182 after 5 and 12.5 Gy. IL-1α levels in fibrosis-resistant (C3H/HeJ) mice increased at days 56 and 182 after 12.5 Gy. These findings demonstrate early and persistent alterations in TNF-α, IL-1α, and IL-1β mRNA levels, even at the lower dose of 5 Gy [271].

Irradiation and inflammation are both associated with increased ROS levels. IR initiates mtROS generation in bone marrow-derived macrophages (BMDMs) in a dose-dependent manner, leading to a pro-inflammatory response. Moreover, radiation-induced mtROS modulates the inflammatory response by affecting the mRNA expression of TNF-α, IL-6, and IL-12p40 in BMDM [272]. Mitochondrial stress activates the cGAS and AIM2 signalling pathways, subsequently initiating inflammatory responses through radiation-induced non-target effects [273]. SIRT3 is involved in persistent radiation-induced damage to the liver. Irradiated *Sirt3*^−/−^ mice show overexpression of inflammatory markers, including IL-6, IL-1β, TGF-β, and lymphoplasmacytic inflammation in the livers [274]. MSC irradiation increased IL-1β levels in a dose-dependent manner. However, treatment of MSCs with resveratrol for 1 h before irradiation activated SIRT1 and caused a decrease in IL-1β expression. At the same time, knockdown of SIRT1 suppresses resveratrol-mediated anti-inflammatory activity [275].

Radiation-induced DNA damage in fibroblasts activates persistent DDR, formation of persistent γH2AX foci, and cell cycle arrest, which are correlated with IL-6 secretion [161]. Partial irradiation of rat lungs initiates cyclic DNA damage and a cyclic pattern of increased expression of IL-1α, IL-1β, IL-6, TNF-α, and TGF-β [276]. 

Atomic bomb survivors exhibit chronic inflammation mediated by the effects of radiation and natural aging, as demonstrated by the changes in cytokine and ROS levels [277]. Special attention has been paid to the investigation of inflammatory status in individuals occupationally exposed to IR. PBMC analysis in astronauts revealed overexpression of TNF-α, IL-1α, and IL-1β genes three days after return to Earth [200]. Healthcare workers exposed to IR showed elevated levels of O_2_•^−^ and three cytokines: IL-6, IL-1α, and MIP-1α. A positive correlation was found between O_2_•^−^ and MIP-1α levels. Workers exposed to high doses of radiation had significantly higher levels of MIP-1α than those exposed to low doses of IR [209].

## 6. Conclusions

In this study, we have delved into the intricate molecular and cellular mechanisms underlying natural aging and the processes induced by ionizing radiation. Our comprehensive analysis has revealed significant parallels between these phenomena. Notably, both aging and radiation exposure manifest genomic instability, characterized by an increased occurrence of double-strand DNA breaks. While most cells with such damage either activate DNA repair mechanisms or undergo apoptosis, unrepaired DSBs resulting from radiation exposure can lead to persistent cellular dysfunction and late radiation effects, such as inflammation.

The inflammatory profile shows similarities between naturally aging cells and those exposed to radiation. The activation of common signaling pathways underscores the convergence of these processes. Through intercellular communication, ionizing radiation and natural cellular senescence have similar effects on neighboring cells, causing persistent changes with chronic consequences. The bystander effect in both natural and radiation-induced aging is caused by exosomes and microRNAs.

Moreover, the expression profile of miRNAs, particularly those regulating the mitochondrial function within the mitomiRs group, presents intriguing parallels between naturally aging cells and those undergoing radiation-induced senescence. Mitochondrial dysfunction emerges as a significant contributor to cellular senescence, impacting cellular energetics, reactive oxygen species generation, mitophagy regulation, and epigenetics.

Epigenetic changes also exhibit striking resemblances, including genome-wide hypomethylation and accumulation of repressive histone marks, irrespective of the initiating factor. Remarkably, radiation-induced alterations in the epigenetic profile demonstrate transgenerational effects, indicating long-lasting consequences.

In the second half of the 20th century, the cellular basis of aging was expressed, noting that by understanding how individual cells age, we will get an idea of how the organism as a whole ages [278]. Hayflick’s experiment also demonstrated that tissue aging may be caused by a gradual loss of cell proliferation, which is necessary to replace damaged cells that naturally accumulate over time [23]. The notion that senescence plays a role in aging has been a subject of speculation for quite some time. However, only recently have researchers been able to confirm this hypothesis. It has taken several decades to develop tools to prove that the buildup of senescent cells contributes to the aging and dysfunction of an organism [16,279,280,281,282]. Cellular senescence occurs in response to many different triggers, many of which have been uncovered in this study. Thus, the accumulation of detrimental features in dividing cells contributes to cellular senescence but may also have broader implications for aging at the organismal level, contributing to the development of age-related pathologies. For example, metabolic dysfunction is associated with aging at the organismal and molecular levels [279]. Other studies have emphasized the connection between SASP, chronic inflammation, and tissue dysfunction, illustrating that cellular senescence is a key contributor to tissue aging. Furthermore, the accumulation of an excessive number of senescent cells has been shown to accelerate aging and contribute to age-related diseases, ultimately leading to organismal aging [280,281].

In light of these findings, it becomes evident that there exists a considerable convergence in the molecular mechanisms underlying natural and radiation-induced aging. This realization opens avenues for the transfer of various gerontological technologies to mitigate the adverse consequences of radiation exposure on populations. While attempts are underway to leverage senolytics for the therapy and prevention of radiation-induced damage, further research is imperative to fully elucidate these processes and develop targeted interventions.

In summary, our study underscores the necessity for comprehensive exploration and understanding of these shared mechanisms, facilitating the development of strategies to alleviate the detrimental effects of radiation exposure and promote healthier aging trajectories.

## Figures and Tables

**Table 1:** Role of miRNAs in cellular senescence (SA-miRNAs).

miRNAs	Expression	Effect	Ref.
miR-34a	↑	Targets *SIRT1* and reduces its expression, leading to the activation of p53 and induction of cellular senescence	[83]
		It is actively expressed in cardiac cells of aged mice, and inhibition or deletion reduces age-associated cardiomyocyte death	[84]
		Increased expression in H2O2-induced premature cellular senescence	[85]
miR-21	↑	Overexpression reduces the replicative lifespan of HUVECs	[86]
miR-146a	↓	Reduced expression in senescent HUVECs	[87]
	↑	Increased expression of senescent lin-BMCs	[88]
miR-29	↑	Rb-dependent increase in expression during replication and induced cellular senescence	[89]
miR-22	↑	Promotes cellular senescence in human fibroblasts and epithelial cells by targeting *CDK6*, *Sp1*, and *SIRT1*	[90]
		Ablation prevented cellular senescence in white adipose tissue (WAT) induced by obesity	[91]
miR-19b	↓	Six models of cellular senescence show reduced expression	[92]
		Reduced expression in age-associated cardiac aging	[93]
miR-26b	↑	Overexpression induces cellular senescence in human epithelial cells and fibroblasts	[94]
miR-320c	↓	Decreased expression with age and participation in juvenile chondrocyte properties by regulating *ADAMTS5* expression	[95]
miR-199a-3p	↑	Increased expression with age; involvement in chondrocyte senescence by regulating *aggrecan*, *COL2*, and *SOX9*	[95]
miR-20a	↓	Six models of cellular senescence show reduced expression	[92]
		Overexpression inhibits stress-induced senescence in WI-38 cells	[96]
miR-155	↓	Reduced expression in Ras-induced senescent WI-38 cells; knockdown increases stress-induced cellular senescence	[96]
miR-210	↑	Overexpression induces senescence in human epithelial cells and fibroblasts	[94]
		Induces the formation of γH2AX foci and ROS in IMR90 cells; increased expression results in an age-related phenotype	[82]
miR-106a	↓	Five models of cellular senescence show reduced expression	[92]
miR-17-92	↓	Increased expression in primary human fibroblasts inhibits Ras-induced cellular senescence	[97]
miR-15a	↓	Involved in the regulation of stress-induced senescence of WI-38 cells	[96]
miR-144	↑	Increased expression in aged erythrocytes from type 2 diabetic patients	[98]
miR-494	↑	Overexpression enhances DNA damage and cellular senescence in IMR90 cells	[82]
miR-449a	↓	Increased expression slows senescence in HUVECs and adipose tissue by targeting p16^Ink4a^, p21^CIP1^, and the PI3K-mTOR signaling pathway	[99]
miR-17	↓	All seven models of cellular senescence show reduced expression	[92]
miR-25	↓	Downregulation in Ras-induced senescent WI-38 cells	[96]
miR-431	↑	Increased expression in both replicative and stress-induced senescent human lung fibroblasts	[96]

**Table 2:** Persistent γH2AX foci after exposure to ionizing radiation.

Cell Type	Radiation	Study Design	Effect of γH2AX	Ref.
Neurons	γ-rays 5 Gy	Whole-body irradiation of mice at postnatal day 3 (P3), P10, and P21. Animals were euthanized at 1, 7, and 120 day(s) and 15 months after irradiation	Consistently demonstrated radiation-induced γH2AX foci or PDDF, in the brains of mice at 120 days and 15 months after irradiation at P3, P10, and P21	[181]
Lymphocytes	γ-rays 0.5–10 Gy	Human G(0)-lymphocytes were irradiated at doses ranging from 0.5 to 10 Gy. The dose response of γH2AX foci was analyzed 24 h, 96 h, 1 week, 2 weeks, and 4 weeks after irradiation	Residual γH2AX foci persisted in human lymphocytes up to 4 weeks after irradiation	[183]
HUVECs	X-rays 1 or 5 Gy	Synchronized G0/G1 phase HUVECs were irradiated with an X-ray dose of 1 or 5 Gy and IRIF were studied from 10 min to 7 days after irradiation	By 7 days after irradiation with 5 Gy, the mean number of γH2AX IRIF per nucleus was still significantly more than in unirradiated cells	[182]

**Table 3:** miRNAs in radiation-induced cellular senescence. Changes in SA-miRNA expression after IR exposure.

miRNAs	Effect	Ref.
miR-34a	Targeting *c-Myc* suppressed its expression, which markedly enhanced IR-induced senescence in human NSCLC cells	[212]
	↑ expression was more than two-fold in thyroid cells shortly after IR irradiation	[213]
	Expression reached a maximum at 3 days in WI-38 cells after exposure to IR, participating in the induction of senescence, and by day 7, the expression level decreased to an insignificant level	[96]
miR-21	↑ expression levels in radiation-induced thymic lymphoma tissue samples in BALB/c mice	[214]
miR-146a	↑ expression levels at 8 h and 24 h in TK6 cells exposed to X-rays	[215]
miR-29a-3p	↑ expression in exosomes during IR-induced fibroblast senescence	[105]
miR-22	↑ expression in rBMSCs after IR irradiation enhanced mtROS production and reduced cell viability	[190]
miR-19b	↓ expression only in IR-induced senescence in WI-38 cells	[96]
miR-26b-5p	↓ expression in blood serum is associated with poor survival in patients with lung adenocarcinoma after radiotherapy	[216]
miR-320a	Linear ↑ in expression in HeLa cells as a function of IR dose and treatment duration	[217]
miR-320b	↓ expression attenuated IR-treatment-induced DNA damage in HCC cells	[218]
miR-199a-3p	Expression in plasma at or below the median abundance at day 6 was associated with decreased survival and early mortality after irradiation in non-human primates (NHP)	[219]
miR-20a	↓ expression levels in IR-induced senescence in WI-38 cells	[96]
miR-155	↓ expression levels in IR-induced senescence in WI-38 cells	[96]
miR-210	Overexpression in OVCAR3 and SKOV3 cancer cell lines reduced their sensitivity to radiotherapy after IR irradiation.	[220]
miR-106a	↓ expression levels in both IR-induced and replicative senescence in WI-38 cells	[96]
miR-17-92	Overexpression significantly increased survival of Z138c cells after IR treatment	[221]
miR-15a	↓ expression levels in IR-induced senescence in WI-38 cells	[96]
miR-144	↑ expression in plasma of irradiated animals on days 3 and/or 7	[222]
miR-494	Differential expression in the blood of mice irradiated Fe^56^	[223]
miR-449a	↑ expression in IR action of LNCaP cells	[224]
miR-17	↓ expression levels in both IR-induced and replicative senescence in WI-38 cells	[96]
miR-25	↓ expression levels in IR-induced senescence in WI-38 cells	[96]
miR-431	↑ expression levels in IR-induced senescence in WI-38 cells	[96]
